# Susceptibility of *Anopheles gambiae s.l.* to the neonicotinoid insecticide clothianidin in eighteen sites located along the south–north transect of Benin

**DOI:** 10.1186/s41182-025-00694-9

**Published:** 2025-02-12

**Authors:** Steve Zinsou Hougbe, Arthur Sovi, Koffi Koumodji, Minassou Juvénal Ahouandjinou, Zul-kifl Affolabi, Linda Towakinou, Saïd Chitou, Andil Agbo-Ola, Filémon Tokponnon, David Mahouton Zoungbédji, Hermann Sagbohan, Casimir Kpanou, Germain Gil Padonou, Lamine Baba-Moussa, Razaki A. Ossé

**Affiliations:** 1 Laboratoire de Biologie et de Typage Moléculaire en Microbiologie (LBTMM), Département de Biochimie et de Biologie Cellulaire (BBC), Université de Abomey-Calavi (UAC), Abomey-Calavi, Benin; 2 Faculté des Sciences et Techniques de l’Université d’Abomey Calavi, Abomey-Calavi, Benin; 3 Faculty of Agronomy, University of Parakou, Parakou, Benin; 4 Faculty of Infectious and Tropical Diseases, The London School of Hygiene and Tropical Medicine, London, UK; 5 Laboratoire des Sciences Animales et Halieutiques, Unité de Recherche en Santé Animale et Biosécurité, Université Nationale d’Agriculture, Kétou, Bénin; 6 École de Gestion et d’Exploitation des Systèmes d’Elevage, Université Nationale d’Agriculture, Kétou, Benin; 7 Centre de Recherche Entomologique de Cotonou, Cotonou, Benin; 8 Ecole Polytechnique d’Abomey-Calavi, Université d’Abomey-Calavi, Abomey-Calavi, Benin

**Keywords:** Clothianidin, Neonicotinoid, Susceptibility, *Anopheles gambiae s.l.*, Benin

## Abstract

**Background:**

The widespread resistance of malaria vectors to traditional neurotoxic insecticides has stimulated the search for new insecticide classes with novel modes of action. For that, the present study was designed to collect data on the susceptibility of field-collected *Anopheles gambiae* sensu lato (*s.l.*) to clothianidin, a neonicotinoid insecticide used in agriculture and that recently received WHO approval for use in indoor residual spraying.

**Methods:**

*An. gambiae s.l.* were collected as larvae and pupae from 18 sites located along the south–north transect of Benin, and reared to adulthood. Female mosquitoes aged 2–5 days were exposed to clothianidin-impregnated papers (2% weight by volume (w/v) of SumiShield™ 50WG dissolved in distilled water). Due to the delayed action of clothianidin, mortality was daily recorded over 7 days. Polymerase chain reaction was used to assess the molecular species composition in the *An. gambiae s.l.* complex and the frequency of knockdown resistance (*kdr*) and insensitive acetylcholinesterase (*Ace-1*^*R*^) mutations.

**Results:**

Mortality rates of field-collected *An. gambiae s.l.* were very high (≥98%) between 2- and 7-day post-exposure, indicating full susceptibility to clothianidin. Molecular species identification revealed the presence of *An. coluzzii* (53.7%), *An. gambiae* sensu stricto (*s.s.*) (42.5%), and *An. arabiensis* (3.8%) in the *An. gambiae s.l.* complex. *kdr* and *Ace-1*^*R*^ mean frequencies were 84% (95% CI 82–86) and 3% (95% CI 2–4) in *An. coluzzii*, and 88% (95% CI 87–90) and 4% (95% CI 3–6) in *An. gambiae s.s*., respectively.

**Conclusions:**

Findings of the present study indicates that *An. gambiae s.l.* populations collected along the north–south transect of Benin remain susceptible to clothianidin. This broadens the portfolio of indoor residual spraying products that the national malaria control programme can deploy to better control pyrethroid-resistant populations of vectors.

**Supplementary Information:**

The online version contains supplementary material available at 10.1186/s41182-025-00694-9.

## Background

In sub-Saharan Africa, indoor residual spraying (IRS) and long-lasting insecticide-treated nets (LLINs) have contributed to reducing malaria mortality and morbidity for several years [1, 2]. In Benin, the first IRS campaign was carried out in the Ouémé department in 2008, using bendiocarb, a carbamate insecticide [3]. Despite the positive impact of this intervention on key entomological indicators of malaria transmission [3–5], the strategy was relocated to the Atacora department, which has a single transmission season, compared with the Ouémé department, characterized by two transmission seasons. This relocation was justified by the fact that bendiocarb has a low remanence that can easily cover the single transmission season observed in the Atacora department, which was not the case in the Ouémé department. Unfortunately, the emergence of bendiocarb resistance in mosquito vector populations [6], has led to its replacement by two formulations of an organophosphate insecticide, pirimiphosmethyl 50 EC and pirimiphos-methyl 300 CS from 2013 to 2019 [7]. To manage insecticide resistance in mosquito vectors, the WHO recommends a management plan based on the use of insecticides with a new mode of action, or a rotation strategy between two or three insecticides, or the combination or mixing of insecticides with different modes of action [8]. Thus, SumiShield® 50WG, containing clothianidin; and Fludora® Fusion, a mixture of clothianidin and deltamethrin, have been developed by Sumitomo Chemical and Bayer Environmental Science, respectively [9, 10]. Clothianidin has a new mode of action to vector control which involves irreversible binding to nicotinic acetylcholine receptors (nAChRs) in the central nervous system of insects. This process interrupts the transmission of nerve impulses, resulting in paralysis, loss of bodily functions, and death [11].

The IRS campaigns conducted in 2020 and 2021 in northern Benin were done with Fludora® Fusion and SumiShield® 50WG, replacing Actellic® 300 CS. However, these products were used without any basic data on the susceptibility of *Anopheles gambiae s.l.* to clothianidin, either in the two target departments or in the 10 other departments of Benin. Instead, the choice of these products was based on data collected in 43 sites located across 16 sub-Saharan African countries, which revealed the susceptibility of *Anopheles* vectors to clothianidin impregnated at the provisional diagnostic dose of 2% w/v in 88% of the sites investigated [11]. There was, however, variability in responses between different sites with some populations showing lower mortality rates than others.

It is, therefore, important to generate data on the susceptibility to clothianidin in populations of *An. gambiae s.l.* from Benin with widespread and close to fixation West African *kdr* mutation* (*conferring pyrethroid and organochlorine resistance), and low frequencies *Ace-1*^*R*^ mutation^#^ (^#^conferring organophosphate and carbamate resistance) [12]. These data will allow to have an overall view of the situation in the country, and serve as a basis for the choice of insecticides for IRS campaigns currently under discussion at the National Malaria Control Program (NMCP).

## Methods

### Study area

**Fig. 1:**
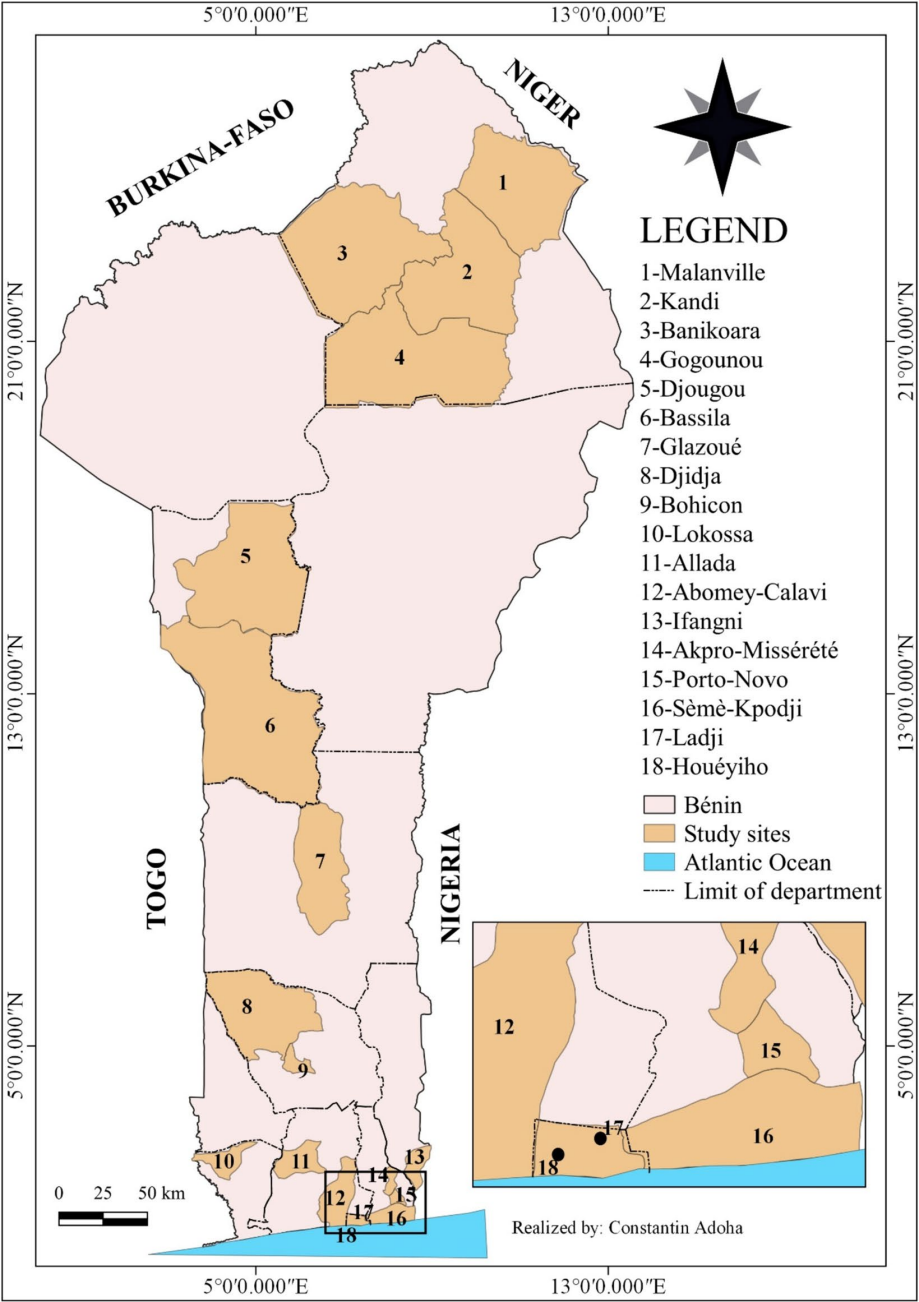
Map of Benin showing the study sites

The study was carried out from January to December 2021 in eighteen (18) sites located along the north–south transect of Benin, a west African country (Fig. 1).

The sites of Ladji, Houeyiho, Ifangni, Akpro-Missérété, Porto-Novo, Sèmè-Kpodji, Abomey-Calavi and Allada selected in the south of the country, are characterized by a traditional food-producing agriculture (cassava, potato, tarot, oil palm), or the practice of market-gardening and fishing due to the proximity of waterways. Those of Bohicon, Djidja, Lokossa and Glazoué selected in the centre of the country, are characterized by a massive cultivation of legumes and cereals including corn, beans and peanuts. These crops are associated with the mass use of chemical fertilizers to increase agricultural yield. Finally, the sites of Malanville, Gogounou, Kandi, Banikoara, Djougou and Bassila selected in the north of the country, are characterized by the cultivation of rice, soybeans and cotton which involves the use of various insecticides and weedkillers.

Overall, the south and centre of the country have a subequatorial climate with two rainy seasons (April to mid-July and September to November) and two dry seasons (December to March and mid-July to August), while the north has a Sudanian climate with a dry season (November to April) and a rainy season (May to October) [13].

### Collections and rearing of immature mosquitoes

During the rainy seasons, mosquito larvae and pupae were collected from breeding sites using a larval dipper. The larvae and pupae were then transported to the insectary and reared to adulthood. Adult mosquitoes were morphologically identified under a binocular microscope using the identification key of Coetzee et al*.* [14]. Unfed females *An. gambiae s.l.* aged 2–5 days were used for clothianidin susceptibility tube tests.

### Susceptibility of *An. gambiae s.l.* to clothianidin

#### Impregnation of Whatman® filter papers

The impregnation of Whatman® filter papers was performed according to the protocol described by Oxborough et al*.* [11]. Papers were cut to 12 cm by 15 cm and impregnated with 13.2 mg of formulated clothianidin, the AI (active ingredient). A stock solution was prepared by diluting in a falcon tube 264 mg of SumiShield® 50WG in 20 ml of distilled water. The mixture was then stirred until the insecticide was completely dissolved. Two milliliters (ml) of the mixed solution were then pipetted evenly onto each filter paper placed on a bed of nails driven at equal height into a wooden board (see video [15]). Each impregnated paper was stored at 4 °C and used within 24 h of treatment. A filter paper treated with 2 ml of distilled water was used as a negative control. The laboratory susceptible strain (Kisumu) was also exposed to clothianidin-impregnated papers for 60 min to validate the test.

### WHO susceptibility tube tests

The susceptibility of field-collected *An. gambiae s.l.* to clothianidin was assessed using WHO tube tests [16], with modifications made to the observation period. Indeed, four replicates each containing 20–25 females *An. gambiae s.l*., aged 2–5 days were exposed to papers impregnated with 2% w/v clothianidin for 60 min. Two replicates with 20–25 females *An. gambiae s.l*. each, were exposed to untreated papers and served as controls. After exposure, mosquitoes were transferred to observation tubes lined with untreated paper and held at 27 °C ± 2 °C and 80% relative humidity with access to 10% (w/v) sugar solution which was changed daily. Mortality was recorded everyday up 7-day post-exposure, to account for the delayed action of clothianidin. Mosquito samples from the WHO susceptibility tube tests were subsequently preserved on silica gel for molecular analyses.

### Molecular analyses

A subsample of mosquitoes (n ≥ 49) were randomly selected from those used in tube tests for PCR analysis.

Molecular species identification within the *An. gambiae s.l.* complex was performed according to the protocol of Santolomazza et al*.* [17]. The reaction medium consisted of 10X buffer, 1.5 mM MgCl2, 0.2 mM dNTPs and 10 pmol of each primer (F6.1a: 5′TCGCCTTAGACCTTGCGTTA3′, R6.1b: 5′CGCTTCAAGAATTCGAGATAC3′). The amplification conditions included an initial denaturation at 95 °C for 5 min, followed by 35 cycles of a final denaturation at 95 °C for 30 s, a hybridization at 54 °C for 1 min, an initial elongation at 72 °C for 1 min, followed by a final elongation of 72 °C for 5 min.

The West African *kdr* mutation was detected according to the protocol of Martinez Torrez et al*.* [18]. The mix was composed of 10X buffer, 1.5 mM MgCl2, 0.2 mM dNTPs, 10 pmol of each primer (D1 5′ATAGATTCCCCGACCATG3′, D2 5′AGACAAGGATGATGAACC3′, D3 5′AATTTGCATTACTTACGACA3′, D4 5′CTGTAGTGATAGGAAATTTA3′), 5 U/µl of Taq polymerase, and mosquito genomic DNA. The amplification conditions were as follows: an initial denaturation of 3 min at 94 °C, followed by 35 cycles including a final denaturation at 94 °C for 30 s, a hybridization of primers at 46 °C for 30 s, an initial elongation at 72 °C for 1 min, followed by a final elongation at 72 °C for 5 min.

The presence of the *Ace-1*^*R*^ mutation was investigated in *Anopheles gambiae s.l.* following the amplification protocol of Weill et al*.* [19]. The reaction medium consisted of a mixture of 10× buffer, 1.5 mM MgCl2, 2.52 mM dNTPs, 10 pmol of each of the primers (MOUSTDIR1 5′CCGGGNGCSACYATGTGGAA3′, MOUSTREV1 5′ACGATMACGTTCTCYTCCGA3′), 5 U/µl of Taq polymerase, and mosquito genomic DNA. The PCR program consisted of the following steps: an initial denaturation of 5 min at 93 °C, 35 cycles including a final denaturation at 93 °C for 1 min, hybridization of primers at 53 °C for 1 min, an initial elongation at 72 °C for 90 s, followed by a final elongation at 72 °C for 10 min. An enzymatic digestion was performed using 1 µl of the restriction enzyme Alu1, and 2 µl of the buffer for 15 μl of the PCR product. An incubation at 37 °C was performed for 8 h.

### Data analyses

Mortality rates to clothianidin were recorded every 24 h after exposure, for 7 days. When mortality of the negative control was greater than 20%, the test was considered invalid and repeated.

The susceptibility of field-collected *An. gambiae s.l.* was interpreted according to WHO criteria [16] as follows:

- Mortality rate ≥ 98%: susceptible mosquito population;

- Mortality rate between 90 and 97%: mosquito population suspected of resistance, need for further investigation;

- Mortality rate < 90%: mosquito population with confirmed resistance.

The frequency of *kdr* and *Ace-1*^*R*^ mutations was calculated by the following formula: F(R) = [2*n*.RR + *n*.RS]/[2(*n*.RR + *n*.RS + *n*.SS)] [20] (*n* = number of mosquito samples with a given genotype).

Confidence intervals of mortality rates, as well as frequencies of resistance mutations were calculated using the binomial test.

All analyses were performed using R software, version 4.1.3.

## Results

### Susceptibility of *An. gambiae s.l.* to clothianidin

**Fig. 2:**
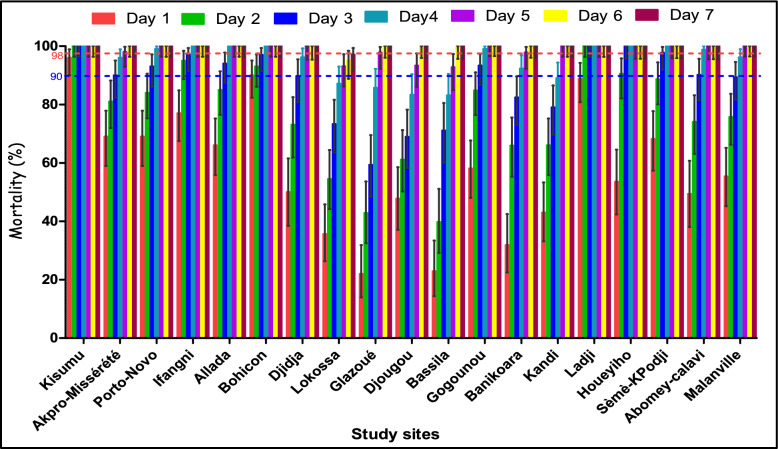
Mortality rates of laboratory susceptible (Kisumu) and field-collected (*Anopheles gambiae* sensu lato) strains after exposure to filter papers impregnated with clothianidin (2% w/v) in World Health Organisation tube tests. The *blue* and *red horizontal dotted lines* indicate the 90 and 98% mortality rates

Mortality of the susceptible Kisumu reference strain was 100% 48 h after exposure to clothianidin-impregnated papers, showing their continued susceptibility (Fig. 2).

With field-collected populations of *An. gambiae s.l*., mortality was ≥98% at all sites after 7 days indicating susceptibility. However, the time to reach this threshold varied between study sites. Thus, susceptibility was observed earlier (between days 2 and 3) in Ladji, Houeyiho, and Sèmè-Kpodji. In the rest of the study sites, it was reached from day 4, with Lokossa being the last to reach it at day 7 post-testing (Fig. 2). Details about the number of tested and dead mosquitoes in each study site over the different observation days were mentioned in the Supplementary Files, Tables S1 and S2.

### Molecular identification of sibling species in *Anopheles gambiae s.l.*

Overall, *An. coluzzii*, *An. gambiae s.s*., and *An. arabiensis* were identified in the *An. gambiae s.l.* complex, and accounted for 53.7% (*n* = 535), 42.5% (*n* = 423), and 3.8% (*n* = 38) of the total number of samples tested (*n* = 996), respectively.

**Fig. 3:**
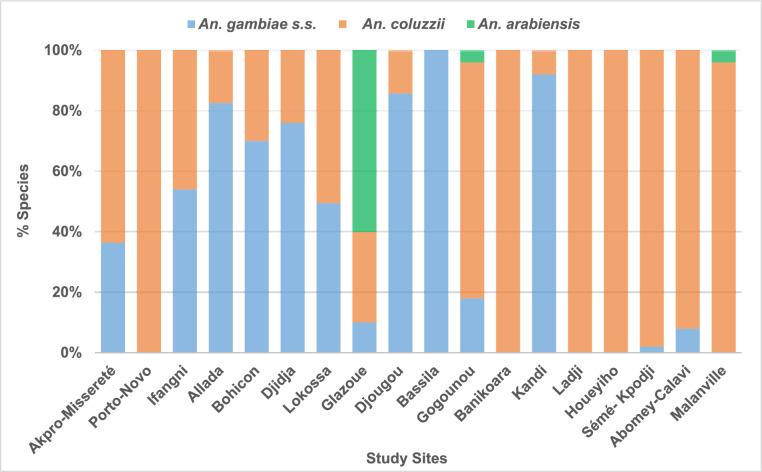
Distribution of molecular species in the *Anopheles gambiae* sensu lato complex at the different study sites

Figure 3 shows the molecular species composition of the *An. gambiae s.l.* complex at each study site. Thus, *An. coluzzii* and *An. gambiae s.s.* were found in sympatry at all sites except Ladji, Bassila, Porto-Novo and Houéyiho. In addition, *An. arabiensis* was identified in low (<8%) and relatively high (60%) proportions at two sites in the north (Gogounou and Malanville), and one site in the centre of the country (Glazoué), respectively (Fig. 3).

### Frequency of the *kdr* and *Ace-1*^*R*^ mutations in *An. coluzzii*, *An. gambiae s.s*. and *An. arabiensis*

In *An. coluzzii*, the frequency of the *kdr* mutation varied between 73% (95% CI 54–87) in Djidja and 100% (95% CI 77–100) in Djougou, with a mean of 84% (95% CI 82–86) across all sites. In *An. gambiae s.s*., it varied from 82% (95% CI 72–90) in Djougou to 100% (95% CI 15–100) in Sémé-Kpodji, with a mean of 88% (95% CI 87–90) across all sites. In *An. arabiensis* it ranged between 75% (95% CI 19–99) in Malanville and Gogounou and 97% (95% CI 87–99) in Glazoué, with a mean of 92% (95% CI 83–97) for the whole study area (Supplementary Files, Table S3). A figure showing the electrophoresis gel of the West African* kdr* mutation is provided in the Supplementary files, Figure F1A.

Regarding the *Ace-1*^*R*^ mutation, its frequency was relatively low at most study sites (<12%), with an average of 3% (95% CI 2–4) in *An. coluzzii,* and 4% in *An. gambiae s.s*. (95% CI 3–6) and *An. arabiensis* (95% CI 1–12) across all sites (Supplementary Files, Table S4). A figure of the electrophoresis gel of the *Ace-1*^*R*^ mutation is in the Supplementary files, Figure F1B.

The low numbers of mosquitoes tested at some sites did not allow for an accurate estimate of mutation frequency (Supplementary Files, Tables S3 and S4).

## Discussion

This study investigated the susceptibility of malaria vector populations collected from different settings along the north–south transect of Benin to the neonicotinoid clothianidin. The results showed that dominant malaria vector species in the *Anopheles gambiae s.l.* complex from these regions remained susceptible to clothiandin. These findings suggest that IRS with clothianidin-based products may be an appropriate strategy to improve the control of malaria transmitted by insecticide-resistant mosquitoes in Benin.

The molecular species composition observed in the present study corroborates the results of the works of Gnanguenon et al. [21], and Koukpo et al. [22]. Indeed, these authors revealed that *An. coluzzii* was predominant, followed by *An. gambiae s.s*., and An. arabiensis* (*found at low frequency) within the *An. gambiae s.l.* complex in Benin. In addition, they observed that *An. arabiensis*, which was originally identified in the north of the country, was increasingly observed in sites in the centre and south of the country (Dassa, Bohicon, and Allada) as is the case in the present study. Indeed, the population of *An. gambiae s.l.* mosquitoes tested in Glazoué (a site in the centre of the country) comprised 60% (30/50) of *An. arabiensis*. The extension of the geographical range of *An. arabiensis* could be due to the combination of two key factors: the zoophilic nature of the species, demonstrated by several studies [23, 24], combined with the increasing movement of livestock from the north to the centre and south of the country associated with the expansion of grazing lands to support the growing population of Benin [25]. Indeed, mosquito larvae from the Glazoué site were mainly collected near small stagnant or very slow-flowing streams used as watering holes for large herds of cattle. Furthermore, the effects of climate change resulting in higher temperatures and lower relative humidity could also contribute to the extension of the species outside its original range [26].

Overall, the *Ace-1*^*R*^ mutation was found at low frequency (≤4%), while the *kdr* mutation had a high frequency (>80%) in the populations of *An. gambiae s.l.* tested. These results corroborate those obtained by Sagbohan et al. [27] in three communes of northern Benin, and suggest that the *kdr* mutation is close to fixation in *An. gambiae s.l.* Despite the presence of resistance mutations in these different mosquito populations, they proved to be fully susceptible to clothianidin (2% w/v), although mortality occurred slowly. Similar results were obtained by Oxborough et al. [11] in 88% (38/43) of the sites investigated across 16 countries in sub-Saharan Africa. This broad susceptibility of *An. gambiae s.l.* to clothianidin could be explained by its new mode of action to which malaria vectors have not had the opportunity to develop resistance. Furthermore, the mortality rates of less than 98% observed with clothianidin in 12% (5/43) of the sites investigated by Oxborough et al*.* [11] cannot automatically be considered as a real sign of resistance, because the protocol used at the time, which is the same as that of the present study, was provisional and yet to be standardised by WHO, which was done later [28]. Indeed, the new WHO standard protocol requires the dilution of clothianidin in the mixture composed of acetone+Mero® (81% rapeseed oil methyl ester). Then, the impregnation of 250 ml glass bottles must be done with 1 ml of the clothianidin solution at a dose of 4 μg/bottle [22], for the evaluation of the susceptibility of *Anopheles* mosquitoes 24 h post-exposure. It should be noted that the dissolution of clothianidin in acetone alone causes crystallization of the insecticide, a phenomenon responsible for very low absorption of the product by the insect and delayed mortality as observed in the study conducted by Fouet et al*.* [29]. This is why the WHO recommended the dissolution of clothianidin in the mixture consisting of the solvent acetone and the surfactant Mero®, which prevents the crystallization of the insecticide and eases the recording of mortality rates 24 h after the test [28]. Furthermore, an experimental hut study conducted in Benin revealed that clothianidin induced overall high mortality rates (87% on mud walls, and 82% on cement walls), with a persistence of approximately 8–9 months on treated walls [30]. The same trend was observed in the community after the implementation of a clothianidin-based IRS in the Alibori and Donga regions, in northern Benin [31]. The present study revealed that mosquito populations in the communes of Djougou, Kandi and Gogounou, which had previously been sprayed with SumiShield® 50WG during the last IRS campaign [31], turned out to be susceptible to clothianidin after a few years. This shows that the different mosquito populations from these IRS communes have not yet developed resistance to clothianidin. Moreover, an observational analysis of data collected in health facilities in Côte d'Ivoire between 2018 and 2022 revealed a decrease in the incidence of malaria after the implementation of clothianidin-based IRS [32]. These numerous positive results support the community use of clothianidin to offer better protection to local populations.

Of note, in the recent studies that showed variability in bioassay results with some populations showing lower mortality rates than others [11, 29], the authors did not dilute clothianidin in the mixture composed of acetone+Mero® as recommended by Tchouakui et al*.* [33] and the WHO. This shows the need for standardizing bioassay protocols, before vector resistance to a given product is asserted.

Several studies have hypothesized that the overuse of insecticides for agricultural activities highly contaminates mosquito breeding sites, which would contribute to the selection of resistance [34, 35]. Thus, the uncontrolled use of neonicotinoids in agriculture for the control of crop pests could cause chronic exposure of mosquito immatures (larvae and pupae) to their residues, which are highly soluble in water [36], thus promoting the emergence of resistance of mosquito vectors to this insecticide class.

It is, therefore, important to raise among farmers awareness about the rational use of products from this class of insecticides to preserve its impact as long as possible in public health. It is also crucial to carefully monitor the level of vector susceptibility to this class of insecticides. This will allow to adapt vector control strategies to avoid operational failures.

The combination of clothianidin-based IRS with next-generation nets that recently showed positive impact in Benin [37], could also substantially improve control of malaria transmission, and be used as pro-active insecticide resistance management strategy.

A potential limitation of the present study is the lack of evaluation of the effect of clothianidin on each of the two major molecular species (*An. coluzzii* and *An. gambiae s.s*.), what was due to the difficulty in isolating each of the two species in the field-collected populations of *An. gambiae s.l*. prior to the susceptibility testing. Moreover, a possible involvement of metabolic genes in the emergence of clothianidin resistance in *An. gambiae* s.l. should be closely monitored as a recent study [38] have detected signs of possible cross resistance involving the GSTe2 gene in *An. funestus* despite its broad susceptibility to clothianidin.

## Conclusions

The results of the present study showed that field-collected populations of *An. gambiae s.l.* collected along the north–south transect of Benin were susceptible to the neonicotinoid insecticide clothianidin. This broadens the portfolio of IRS products that the NMCP can deploy to improve control of pyrethroid-resistant mosquito populations.

## Supplementary Information

Additional file 1.

## Data Availability

No datasets were generated or analysed during the current study.

## Abbreviations

IRS: Indoor residual spraying
LLINs: Long-lasting insecticide-treated mosquito net
NMCP: National Malaria Control Program
*An. gambiae*: *Anopheles gambiae*
WHO: World Health Organization
