# Supplementary material for: Susceptibility of Anopheles gambiae s.l. to the neonicotinoid insecticide clothianidin in eighteen sites located along the south–north transect of Benin
Source: Trop Med Health. 2025 Feb 12;53:21. doi: 10.1186/s41182-025-00694-9 (PMC11816534; doi:10.1186/s41182-025-00694-9)
Supplement: Supplementary file 1 — Additional file 1. [file 41182_2025_694_MOESM1_ESM.docx]

**Supplementary Files**

Figure F1: Electrophoresis gel for West African *kdr* (A) and *Ace-1^R^* (B) mutations


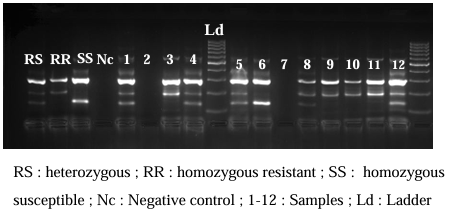


**A**


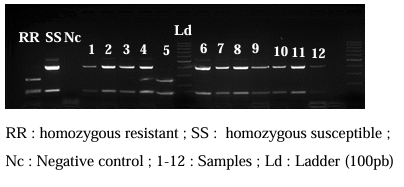


**B**

**Table S1:** Mortality rates of laboratory susceptible (Kisumu) and field-collected (*Anopheles gambiae* sensu lato) strains after exposure to untreated and and clothianidin (2% w/v) impregnated papers in World Health Organisation tube tests (Part 1).

| Districts/Strain | Total tested | Day 1 | Day 2 | Day 3 | Day 4 | Day 5 | Day 6 | Day 7 |
| --- | --- | --- | --- | --- | --- | --- | --- | --- |
|  |  | n (%) (95%CI) | n (%) (95%CI) | n (%) (95%CI) | n (%) (95%CI) | n (%) (95%CI) | n (%) (95%CI) | n (%) (95%CI) |
| Kisumu | 100 | 96 (96) (89.4-98.7) | 100 (100) (95.3-100) | 100 (100) (95.3-100) | 100 (100) (95.3-100) | 100 (100) (95.3-100) | 100 (100) (95.3-100) | 100 (100) (95.3-100) |
|  |  |  |  |  |  |  |  |  |
| Akpro-Missérété | 99 | 69 (69.6) (59.5-78.3) | 81 (81.8) (72.5-88.5) | 90 (90.6) (83-95.4) | 96 (96.9) (90.7-99.2) | 98 (98.9) (93.6-99.9) | 99 (100) (95.3-100) | 99 (100) (95.3-100) |
| Control | 50 | 0 (0) (0-8.9) | 0 (0) (0-8.9) | 0 (0) (0-8.9) | 1 (2) (0.1-12.0) | 1 (2) (0.1-12.0) | 1 (2) (0.1-12.0) | 1 (2) (0.1-12.0) |
|  |  |  |  |  |  |  |  |  |
| Porto-Novo | 90 | 62 (69) (58.1-78) | 76 (84) (74.9-90.9) | 84 (93) (85.5-97.2) | 89 (99) (93-99.9) | 90 (100) (94.8-100) | 90 (100) (94.8-100) | 90 (100) (94.8-100) |
| Control | 50 | 0 (0) (0-8.9) | 0 (0) (0-8.9) | 0 (0) (0-8.9) | 0 (0) (0-8.9) | 0 (0) (0-8.9) | 0 (0) (0-8.9) | 0 (0) (0-8.9) |
|  |  |  |  |  |  |  |  |  |
| Ifangni | 88 | 68 (77.2) (66.8-85.2) | 84 (95.4) (88.1-98.5) | 85 (96.5) (89.6-99.1) | 88 (100) (94.7-100) | 88 (100) (94.7-100) | 88 (100) (94.7-100) | 88 (100) (94.7-100) |
| Control | 50 | 0 (0) (0-8.9) | 0 (0) (0-8.9) | 0 (0) (0-8.9) | 0 (0) (0-8.9) | 0 (0) (0-8.9) | 0 (0) (0-8.9) | 0 (0) (0-8.9) |
|  |  |  |  |  |  |  |  |  |
| Allada | 96 | 66 (68.7) (58.3-77.6) | 85 (88.5) (80-93.8) | 94 (97.9) (91.9-99.6) | 96 (100) (95.2-100) | 96 (100) (95.2-100) | 96 (100) (95.2-100) | 96 (100) (95.2-100) |
| Control | 50 | 0 (0) (0-8.9) | 0 (0) (0-8.9) | 0 (0) (0-8.9) | 0 (0) (0-8.9) | 0 (0) (0-8.9) | 0 (0) (0-8.9) | 0 (0) (0-8.9) |
|  |  |  |  |  |  |  |  |  |
| Bohicon | 87 | 78 (89.6) (80.8-94.8) | 81 (93.1) (85-97.1) | 85 (97.7) (91.1-99.6) | 87 (100) (94.7-100) | 87 (100) (94.7-100) | 87 (100) (94.7-100) | 87 (100) (94.7-100) |
| Control | 50 | 0 (0) (0-8.9) | 0 (0) (0-8.9) | 1 (2) (0.1-12.0) | 1 (2) (0.1-12.0) | 1 (2) (0.1-12.0) | 1 (2) (0.1-12.0) | 1 (2) (0.1-12.0) |
|  |  |  |  |  |  |  |  |  |
| Djidja | 78 | 39 (50) (39.1-60.8) | 58 (74.3) 63-83.2) | 70 (89.7) (80.2-95.1) | 76 (97.4) (90.1-99.5) | 78 (100) (94.1-100) | 78 (100) (94.1-100) | 78 (100) (94.1-100) |
| Control | 50 | 0 (0) (0-8.9) | 0 (0) (0-8.9) | 0 (0) (0-8.9) | 1 (2) (0.1-12.0) | 1 (2) (0.1-12.0) | 1 (2) (0.1-12.0) | 1 (2) (0.1-12.0) |
|  |  |  |  |  |  |  |  |  |
| Lokossa | 101 | 36 (35.6) (26.5-45.8) | 56 (55.4) (45.2-65.2) | 75 (74.2) (64.4-82.2) | 89 (88.1) (79.7-93.4) | 95 (94.) (87-97.5) | 98 (97) (90.9-99.2) | 100 (99) (93.8-99.9) |
| Control | 50 | 0 (0) (0-8.9) | 0 (0) (0-8.9) | 0 (0) (0-8.9) | 1 (2) (0.1-12.0) | 1 (2) (0.1-12.0) | 1 (2) (0.1-12.0) | 1 (2) (0.1-12.0) |
|  |  |  |  |  |  |  |  |  |
| Glazoue | 91 | 20 (21.9) (14.2-32.1) | 39 (42.8) (32.6-53.6) | 56 (61.5) (50.7-71.3) | 78 (85.7) (76.4-91.8) | 89 (97.8) (91.5-99.6) | 91 (100) (94.9-100) | 91 (100) (94.9-100) |
| Control | 50 | 0 (0) (0-8.9) | 0 (0) (0-8.9) | 1 (2) (0.1-12.0) | 1 (2) (0.1-12.0) | 1 (2) (0.1-12.0) | 1 (2) (0.1-12.0) | 1 (2) (0.1-12.0) |
|  |  |  |  |  |  |  |  |  |
| Djougou | 90 | 42 (46.6) (36.1-57.4) | 55 (61.1) (50.2-71) | 62 (68.8) (58.1-78) | 75 (83.3) (73.6-90) | 84 (93.3) (85.5-97.2) | 90 (100) (94.8-100) | 90 (100) (94.8-100) |
| Control | 50 | 0 (0) (0-8.9) | 0 (0) (0-8.9) | 0 (0) (0-8.9) | 1 (2) (0.1-12.0) | 1 (2) (0.1-12.0) | 1 (2) (0.1-12.0) | 1 (2) (0.1-12.0) |

n: number of dead mosquitoes, %: mortality rate, CI: confidence interval

**Table S2:** Mortality rates of laboratory susceptible (Kisumu) and field-collected (*Anopheles gambiae* sensu lato) strains after exposure to untreated and and clothianidin (2% w/v) impregnated papers in World Health Organisation tube tests (Part 2).

| Districts/Strain | Total tested | Day 1 | Day 2 | Day 3 | Day 4 | Day 5 | Day 6 | Day 7 |
| --- | --- | --- | --- | --- | --- | --- | --- | --- |
|  |  | n (%) (95%CI) | n (%) (95%CI) | n (%) (95%CI) | n (%) (95%CI) | n (%) (95%CI) | n (%) (95%CI) | n (%) (95%CI) |
| Bassila | 83 | 19 (22.8) (14.6-33.6) | 34 (40.9) (30.4-52.3) | 59 (71) (59.9-80.2) | 69 (83.1) (72.9-90.1) | 77 (92.7) (84.3-97) | 83 (100) (94.4-100) | 83 (100) (94.4-100) |
| Control | 50 | 0 (0) (0-8.9) | 0 (0) (0-8.9) | 1 (2) (0.1-12.0) | 1 (2) (0.1-12.0) | 1 (2) (0.1-12.0) | 1 (2) (0.1-12.0) | 1 (2) (0.1-12.0) |
|  |  |  |  |  |  |  |  |  |
| Gogounou | 105 | 61 (58) (48-67.5) | 90 (85.7) (77.2-91.5) | 98 (93.3) (86.2-97) | 104 (99) (94-99) | 105 (100) (95.6-100) | 105 (100) (95.6-100) | 105 (100) (95.6-100) |
| Control | 50 | 0 (0) (0-8.9) | 0 (0) (0-8.9) | 0 (0) (0-8.9) | 1 (2) (0.1-12.0) | 1 (2) (0.1-12.0) | 1 (2) (0.1-12.0) | 1 (2) (0.1-12.0) |
|  |  |  |  |  |  |  |  |  |
| Banikoara | 91 | 30 (32.9) (23.6-43.7) | 60 (65.9) (55.1-75.3) | 77 (84.6) (75 -91) | 84 (92.3) (84.2-96.5) | 87 (95.6) (88.5-98.5) | 91 (100) (94.9-100) | 91 (100) (94.9-100) |
| Control | 50 | 0 (0) (0-8.9) | 0 (0) (0-8.9) | 0 (0) (0-8.9) | 0 (0) (0-8.9) | 1 (2) (0.1-12.0) | 1 (2) (0.1-12.0) | 1 (2) (0.1-12.0) |
|  |  |  |  |  |  |  |  |  |
| Kandi | 100 | 43 (43) (33.2-53.2) | 66 (66) (55.7-74.9) | 79 (79) (69.4-86.2) | 89 (89) (80.7-94.1) | 100 (100) (95.3-100) | 100 (100) (95.3-100) | 100 (100) (95.3-100) |
| Control | 50 | 0 (0) (0-8.9) | 0 (0) (0-8.9) | 0 (0) (0-8.9) | 1 (2) (0.1-12.0) | 1 (2) (0.1-12.0) | 1 (2) (0.1-12.0) | 1 (2) (0.1-12.0) |
|  |  |  |  |  |  |  |  |  |
| Ladji | 98 | 87 (88.7) (80.4-93.9) | 98 (100) (95.2-100) | 98 (100) (95.2-100) | 98 (100) (95.2-100) | 98 (100) (95.2-100) | 98 (100) (95.2-100) | 98 (100) (95.2-100) |
| Control | 50 | 0 (0) (0-8.9) | 0 (0) (0-8.9) | 1 (2) (0.1-12.0) | 1 (2) (0.1-12.0) | 1 (2) (0.1-12.0) | 1 (2) (0.1-12.0) | 1 (2) (0.1-12.0) |
|  |  |  |  |  |  |  |  |  |
| Houeyiho | 84 | 45 (53.5) (42.4-64.4) | 76 (90.4) (81.5-95.5) | 84 (100) (94.5-100) | 84 (100) (94.5-100) | 84 (100) (94.5-100) | 84 (100) (94.5-100) | 84 (100) (94.5-100) |
| Control | 50 | 0 (0) (0-8.9) | 0 (0) (0-8.9) | 0 (0) (0-8.9) | 0 (0) (0-8.9) | 0 (0) (0-8.9) | 0 (0) (0-8.9) | 0 (0) (0-8.9) |
|  |  |  |  |  |  |  |  |  |
| Sèmè-Kpodji | 88 | 60 (68.1) (57.2-77.4) | 78 (88.6) (79.6-94.1) | 86 (97.7) (91.2-99.6) | 88 (100) (94.7-100) | 88 (100) (94.7-100) | 88 (100) (94.7-100) | 88 (100) (94.7-100) |
| Control | 50 | 0 (0) (0-8.9) | 0 (0) (0-8.9) | 0 (0) (0-8.9) | 0 (0) (0-8.9) | 0 (0) (0-8.9) | 0 (0) (0-8.9) | 0 (0) (0-8.9) |
|  |  |  |  |  |  |  |  |  |
| Abomey-calavi | 81 | 40 (49.3) (38.1-60.6) | 60 (74) (62.9-82.8) | 73 (90.1) (80.9-95.3) | 80 (98.7) (92.3-99.9) | 81 (100) (94.3-100) | 81 (100) (94.3-100) | 81 (100) (94.3-100) |
| Control | 50 | 0 (0) (0-8.9) | 0 (0) (0-8.9) | 1 (2) (0.1-12.0) | 1 (2) (0.1-12.0) | 2 (4) (0.7-14.9) | 2 (4) (0.7-14.9) | 2 (4) (0.7-14.9) |
|  |  |  |  |  |  |  |  |  |
| Malanville | 103 | 57 (55.3) (45.2-65) | 78 (75.7) (66.1-83.3) | 92 (89.3) (81.3-94.2) | 99 (96.1) (89.7-98.7) | 103 (100) (95.5-100) | 103 (100) (95.5-100) | 103 (100) (95.5-100) |
| Control | 50 | 0 (0) (0-8.9) | 0 (0) (0-8.9) | 0 (0) (0-8.9) | 1 (2) (0.1-12.0) | 1 (2) (0.1-12.0) | 1 (2) (0.1-12.0) | 1 (2) (0.1-12.0) |

n: number of dead mosquitoes, %: mortality rate, CI: confidence interval

**Table S3: Frequency of the *kdr* mutation in *An. coluzzii*, *An. gambiae* s.s. and *An. arabiensis***

| Sites | **Species** | **N tested** | ***1014F*** | ***1014L*** | ***1014L*** | **Freq (%)** | **95% CI** |
| --- | --- | --- | --- | --- | --- | --- | --- |
|  |  |  | ***1014F*** | ***1014F*** | ***1014L*** |  |  |
| Akpro-Misséreté | *An. coluzzii* | 63 | 45 | 14 | 4 | 83 | 75 - 89 |
|  | *An. gambiae s.s.* | 36 | 28 | 5 | 3 | 85 | 74 - 92 |
| Porto-Novo | *An. coluzzii* | 50 | 39 | 7 | 4 | 85 | 76 - 91 |
| Ifangni | *An. coluzzii* | 23 | 17 | 4 | 2 | 83 | 69 - 92 |
|  | *An. gambiae s.s.* | 27 | 21 | 4 | 2 | 85 | 73 - 93 |
| Allada | *An. coluzzii* | 12 | 8 | 4 | 0 | 83 | 63 - 95 |
|  | *An. gambiae s.s.* | 38 | 32 | 5 | 1 | 91 | 82 - 96 |
| Bohicon | *An. coluzzii* | 26 | 19 | 7 | 0 | 87 | 74 - 94 |
|  | *An. gambiae s.s.* | 24 | 16 | 8 | 0 | 83 | 70 - 93 |
| Djidja | *An. coluzzii* | 15 | 10 | 2 | 3 | 73 | 54 - 87 |
|  | *An. gambiae s.s.* | 35 | 27 | 6 | 2 | 86 | 75 - 92 |
| Lokossa | *An. coluzzii* | 50 | 37 | 8 | 5 | 82 | 73 - 89 |
|  | *An. gambiae s.s.* | 49 | 43 | 5 | 1 | 93 | 86 - 97 |
| Glazoue | *An. coluzzii* | 15 | 9 | 5 | 1 | 77 | 57 - 90 |
|  | *An. gambiae s.s.* | 5 | 5 | 0 | 0 | 100 | 69 - 100 |
|  | *An. arabiensis* | 30 | 28 | 2 | 0 | 97 | 87 - 99 |
| Djougou | *An. coluzzii* | 7 | 7 | 0 | 0 | 100 | 77 - 100 |
|  | *An. gambiae s.s.* | 42 | 31 | 7 | 4 | 82 | 72 - 90 |
| Bassila | *An. gambiae s.s.* | 50 | 39 | 8 | 3 | 86 | 78 - 92 |
| Gogounou | *An. coluzzii* | 39 | 31 | 5 | 3 | 86 | 76 - 93 |
|  | *An. gambiae s.s.* | 9 | 8 | 1 | 0 | 94 | 73 - 100 |
|  | *An. arabiensis* | 2 | 1 | 1 | 0 | 75 | 19 - 99 |
| Banikoara | *An. coluzzii* | 6 | 4 | 2 | 0 | 83 | 52 - 98 |
|  | *An. gambiae s.s.* | 43 | 40 | 2 | 1 | 95 | 89 - 99 |
| Kandi | *An. gambiae s.s.* | 46 | 37 | 7 | 2 | 88 | 79 - 93 |
|  | *An. arabiensis* | 4 | 2 | 2 | 0 | 75 | 34 - 96 |
| Ladji | *An. coluzzii* | 50 | 38 | 10 | 2 | 86 | 77 - 92 |
| Houeyiho | *An. coluzzii* | 50 | 38 | 10 | 2 | 86 | 77 - 92 |
| Sémé-Kpodji | *An. coluzzii* | 49 | 37 | 12 | 0 | 88 | 79 - 93 |
|  | *An. gambiae s.s.* | 1 | 1 | 0 | 0 | 100 | 15 - 100 |
| Abomey-calavi | *An. coluzzii* | 46 | 34 | 8 | 4 | 83 | 73 - 89 |
|  | *An. gambiae s.s.* | 4 | 3 | 1 | 0 | 88 | 47 - 99 |
| Malanville | *An. coluzzii* | 48 | 34 | 10 | 4 | 81 | 72 - 88 |
|  | *An. arabiensis* | 2 | 1 | 1 | 0 | 75 | 19 - 99 |
| Total | *An. coluzzii* | 440 | 339 | 78 | 23 | 84 | 82 - 86 |
|  | *An. gambiae s.s.* | 384 | 302 | 65 | 17 | 88 | 87 - 90 |
|  | *An. arabiensis* | 38 | 32 | 6 | 0 | 92 | 83 - 97 |

N: total number, *An : Anopheles*, Freq: frequency, CI: Confidence interval

**Table S4: Frequency of the *Ace-1^R^* mutation in *An. coluzzii*, *An. gambiae* s.s. and *An. arabiensis***

| Communes | Species | N tested | ***119S*** | ***119G*** | ***119G*** | **Freq (%)** | **95%CI** |
| --- | --- | --- | --- | --- | --- | --- | --- |
|  |  |  | ***119S*** | ***119S*** | ***119G*** |  |  |
| Akpro-Missereté | *An. coluzzii* | 63 | 0 | 6 | 57 | 5 | 2 - 10 |
|  | *An. gambiae s.s.* | 36 | 0 | 0 | 36 | 0 | 0 - 5 |
| Porto-Novo | *An. coluzzii* | 50 | 0 | 3 | 47 | 3 | 1 - 9 |
| Ifangni | *An. coluzzii* | 23 | 0 | 3 | 20 | 7 | 1 - 18 |
|  | *An. gambiae s.s.* | 27 | 0 | 3 | 24 | 6 | 1 - 15 |
| Allada | *An. coluzzii* | 12 | 0 | 1 | 11 | 4 | 0 - 21 |
|  | *An. gambiae s.s.* | 38 | 0 | 2 | 36 | 3 | 0 - 9 |
| Bohicon | *An. coluzzii* | 26 | 0 | 2 | 24 | 7 | 1 - 18 |
|  | *An. gambiae s.s.* | 24 | 0 | 1 | 23 | 8 | 0 - 38 |
| Djidja | *An. coluzzii* | 35 | 0 | 1 | 34 | 1 | 0 - 7 |
|  | *An. gambiae s.s.* | 50 | 0 | 2 | 48 | 2 | 0 - 7 |
| Lokossa | *An. coluzzii* | 50 | 0 | 2 | 48 | 2 | 0 - 7 |
|  | *An. gambiae s.s.* | 49 | 0 | 7 | 42 | 7 | 3 - 14 |
| Glazoue | *An. coluzzii* | 15 | 0 | 1 | 14 | 3 | 0 - 17 |
|  | *An. gambiae s.s.* | 5 | 0 | 0 | 5 | 0 | 0 - 30 |
|  | *An. arabiensis* | 30 | 0 | 3 | 27 | 5 | 1 - 13 |
| Djougou | *An. coluzzii* | 7 | 0 | 0 | 7 | 0 | 0 - 23 |
|  | *An. gambiae s.s.* | 42 | 0 | 4 | 38 | 5 | 1 - 12 |
| Bassila | *An. gambiae s.s.* | 50 | 0 | 7 | 43 | 7 | 3 - 14 |
| Gogounou | *An. coluzzii* | 39 | 0 | 7 | 32 | 9 | 4 - 18 |
|  | *An. gambiae s.s.* | 9 | 0 | 0 | 9 | 0 | 0 - 19 |
|  | *An. arabiensis* | 2 | 0 | 0 | 2 | 0 | 0 - 60 |
| Banikoara | *An. coluzzii* | 6 | 0 | 1 | 5 | 8 | 0 - 38 |
|  | *An. gambiae s.s.* | 43 | 0 | 8 | 35 | 9 | 4 - 18 |
| Kandi | *An. gambiae s.s.* | 46 | 0 | 1 | 45 | 1 | 0 - 5 |
|  | *An. arabiensis* | 4 | 0 | 0 | 4 | 0 | 0 - 36 |
| Ladji | *An. coluzzii* | 50 | 0 | 0 | 50 | 0 | 0 - 3 |
| Houeyiho | *An. coluzzii* | 50 | 0 | 0 | 50 | 0 | 0 - 3 |
| Sémé-Kpodji | *An. coluzzii* | 49 | 0 | 1 | 48 | 1 | 0.03 - 5 |
|  | *An. gambiae s.s.* | 1 | 0 | 0 | 1 | 0 | 0 - 84 |
| Abomey-calavi | *An. coluzzii* | 46 | 0 | 2 | 44 | 2 | 0.3 - 7 |
|  | *An. gambiae s.s.* | 4 | 0 | 1 | 3 | 12 | 0.3 - 52 |
| Malanville | *An. coluzzii* | 48 | 0 | 4 | 44 | 4 | 1 - 10 |
|  | *An. arabiensis* | 2 | 0 | 0 | 2 | 0 | 0 - 60 |
| Total | *An. coluzzii* | 569 | 0 | 34 | 535 | 3 | 2 - 4 |
|  | *An. gambiae s.s.* | 424 | 0 | 36 | 388 | 4 | 3 - 6 |
|  | *An. arabiensis* | 38 | 0 | 3 | 35 | 4 | 1 - 12 |

N: total number, *An: Anopheles*, Freq: frequency, CI: Confidence interval
